# Comparison of Novel Sandfly Control Interventions: A Pilot Study in Bangladesh

**DOI:** 10.4269/ajtmh.20-0997

**Published:** 2021-10-25

**Authors:** Debashis Ghosh, Abdul Alim, M. Mamun Huda, Christine M. Halleux, Md. Almahmud, Piero L. Olliaro, Greg Matlashewski, Axel Kroeger, Dinesh Mondal

**Affiliations:** ^1^ Nutrition and Clinical Services Division, International Centre For Diarrhoeal Disease Research, Bangladesh (icddr,b), 68 Shaheed Taj Uddin Ahmed Sarani, Mohakhali, Dhaka-1212, Bangladesh;; ^2^ UNICEF/UNDP/World Bank/World Health Organization Special Programme for Research and Training in Tropical Diseases (TDR), World Health Organization, Geneva, Switzerland;; ^3^ Centre for Tropical Medicine and Global Health, Nuffiled Department of Medicine, University of Oxford, Oxford, United Kingdom;; ^4^ Department of Microbiology and Immunology, McGill University, Montreal, Canada;; ^5^ University Medical Centre Freiburg, Centre for Medicine and Society, Freiburg, Germany

## Abstract

In this pilot comparative study, we investigated and compared the effects of existing vector control tools on sandfly densities and mortality to inform and support the National Kala-azar Elimination Program (NKEP). The interventions included insecticidal wall painting (IWP), reduced-coverage insecticidal durable wall lining (DWL), insecticide-impregnated bednets (ITN), and indoor residual spraying with deltamethrin (IRS). Sakhua union with seven villages was the study area, which was the most highly endemic visceral leishmaniasis union in Trishal upazila, Bangladesh. Each cluster containing the different interventions included approximately 50 households. Study methods included random selection of clusters, collection of sandfly by CDC light trap and manual aspirator to determine sandfly density, and sandfly mortality determined by WHO cone bioassay test. Trained field research assistants interviewed household heads using structured questionnaires for sociodemographic information, as well as safety and acceptability of the interventions. Descriptive and analytical statistical methods measured interventions’ effect and its duration on sandfly density reduction and mortality. We measured the relative efficacy of IWP on sandfly control against DWL, ITN, and IRS by the difference-in-difference regression model. We found that existing interventions were effective and safe for sandfly control with different duration of effect and acceptability. The relative efficacy of IWP for sandfly reduction varied by –59% to –91%, –75% to –81%, and –30% to –104% compared with DWL, ITN, and IRS, respectively, at different time points during the 12-month follow-up. These study results will guide the NKEP for selection of sandfly control tool(s) in its subsequent consolidation and maintenance phases.

## INTRODUCTION

Visceral leishmaniasis (VL) or kala-azar (KA) is a vectorborne disease caused by *Leishmania donovani* and is transmitted by female *Phlebotomus argentipes* sandflies. In the Indian subcontinent, the disease is endemic, and its presence has been documented since the 19th century where the first reported VL outbreak was in 1824 in the territory of current Bangladesh. During this period, VL caused the deaths of 75,000 people over 3 years.^1^ VL peaks periodically, possibly when herd immunity (which is yet to be defined) wanes.^2^

Nevertheless, the ongoing National KA elimination program (NKEP) demonstrates that the VL incidence can be reduced using existing tools for diagnosis, treatment, and vector control with the government’s commitment and support from development partners.^3^ The NKEP has four phases: the preparatory phase, followed by attack, consolidation, and maintenance phases. The target of the attack phase is to reduce VL incidence to less than 1 case per 10,000 people at the upazila, block, and district levels, respectively, in Bangladesh, India, and Nepal.^4^ Nepal and Bangladesh achieved the elimination target of the attack phase in 2013 and 2016, respectively. India is very close as only 6% of the blocks have a VL incidence of > 1 per 10,000 people.^5^

Early detection and management of VL cases, vector control, disease surveillance, community participation and partnership and operational research were the key elements of the attack phase. Although the strategies for subsequent phases are yet to be defined, effective sandfly control is required for the success of the subsequent phases. During the malaria eradication era from 1960 to 1970, VL disappeared from Bangladesh.^1^ The key intervention was indoor insecticide residual spraying (IRS) using DDT for malaria control, and sandfly control was a collateral benefit of the malaria eradication program. This historical evidence, together with evidence for sandfly control with IRS using deltamethrin, alpha-cypermethrin, and DDT on the Indian subcontinent placed IRS as the major sandfly control method of the VL elimination initiative.^6^^,^^7^ IRS, however, is expensive, requires trained personnel to deliver it, requires procuring and maintaining equipment and supplies, needs to be repeated at least twice a year, and has short duration of efficacy. For all these reasons, IRS may not be sustainable in the long run. In Bangladesh, insecticide-treated materials such as commercial insecticide-treated bednets (ITN), slow-release insecticide tablets for impregnation of existing bednets, and durable wall lining (DWL) have also been found to be effective for sandfly control.^8^^,^^9^ However, in India, commercial insecticide-treated bednets did not work, and in Nepal results varied in different studies.^6^^,^^10^ As in Bangladesh, DWL was also effective in India.^11^ Insecticidal wall painting (IWP), a new tool for vector control, has been found to be effective for mosquito control in Africa^12^^,^^13^ and more recently in Nepal for sandfly control.^14^ Taken together, there is a need to investigate the efficacy of IWP compared with other interventions in Bangladesh to inform the NKEP about the best approaches for sandfly control.

In this study, we compared the performance of IWP against existing sandfly control tools including DWL, ITN, and IRS.

The trial ID is ClinicalTrials.gov ID: NCT03269006.

## METHODS

### Study areas, duration, and design.

**Figure 1.**
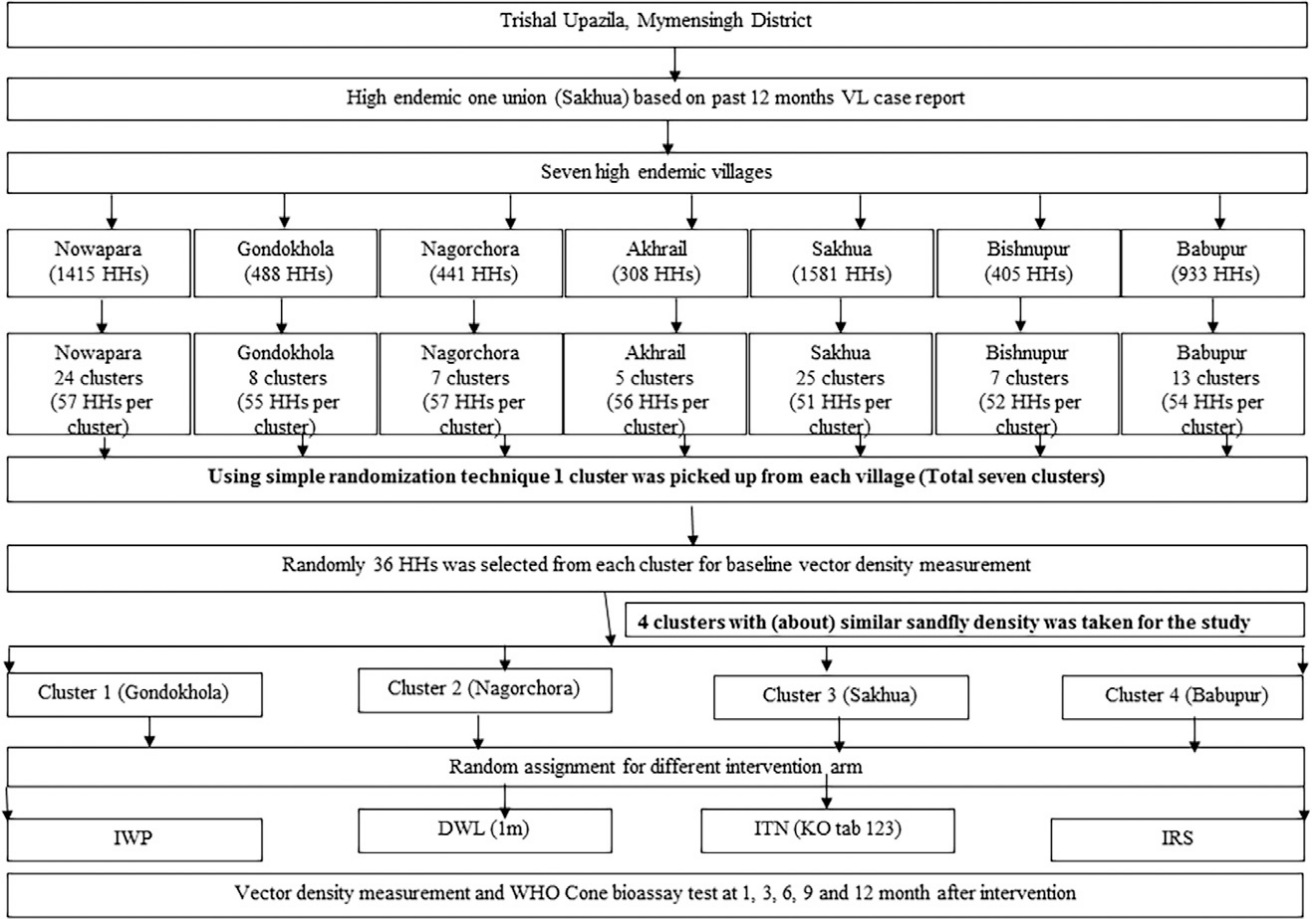
Study design. DWL = durable wall lining containing deltamethrin; HH = households; IRS = indoor residual spraying with deltamethrin; ITN = impregnation of existing bednets with KO TAB 1-2-3; IWP = insecticidal wall painting.

The study was a comparative study carried out from November 2015 to January 2017 in the Trishal upazila of Mymensingh. Trishal has 12 unions (a union has several villages with a total average population of 25,000 people) in which five were VL endemic. We selected seven villages the Sakua union for this study because it had the highest VL burden based on the previous 12 months hospital (Trishal Upazila Health Complex) reports. We divided each village into clusters, each cluster with approximately 50 households (HHs). One cluster from each village was randomly selected, from which we randomly selected 36 HHs for conducting entomological activities. Baseline sandfly measurements (sandfly density) were determined in all seven clusters 2 to 4 weeks before the interventions. Of these seven clusters, four were selected randomly, and interventions were also allocated in the selected four clusters randomly (one intervention for each cluster) where IWP was the intervention of interest for comparison with DWL, ITN, and IRS (Figure 1). Trained FRAs interviewed HH heads through structured questionnaire for collection of socioeconomic demographics and other HH information regarding safety and acceptability of the interventions.

### Interventions.

#### IWP.

Experts from Inesfly, Valencia, Spain, trained field research assistants (FRAs) in painting of different type of HH walls following manufacturer’s standard operating procedures. FRAs painted 56 HHs under the IWP study arm with Inesfly 5AIGRNG TM containing alphacypermethrin 0.7%, D-allethin 1.0%, and pyriproxyphen (0.063%) with a coverage of 2.43 m^2^. The formulation is vinyl paint with an aqueous base, with the active ingredients CaCO_3_ and resin microcapsules, allowing a gradual release of active ingredients. Microcapsules range from one to several hundred micrometers in size. Once painted, the expected concentration of insecticide per square meter surface area of the wall should be 1.225, 1.75, and 0.11 g/m^2^, respectively, for alphacypermethrin, D-allethin, and pyriproxyphen.

#### DWL.

Trained FRAs installed DWL up to 1-m height from floor (reduced wall surface coverage) in the indoor walls of the main room in 55 HHs in this arm. The commercial DWL is deltamethrin-impregnated DWL containing deltamethrin at 170 mg a.i./m^2^.

#### KO TAB 1-2-3 ITN.

Ninety-five existing bednets in 52 HHs in the ITN arm were impregnated following manufacturer’s instruction (KO TAB 1-2-3 containing 0.4 g deltamethrin in a 1.6-g tablet and a chemical binder (Bayer [Ply] Ltd. Reg. No. 1968/011192/07, Isando, South Africa).

#### IRS.

54 HHs received IRS by the deltamethrin 5 WP (Tagros Chemical India Ltd., Chennai, India) and sprayed up to 6 feet (180 cm) of the indoor walls by the research team at month zero.

The NKEP conducted routine IRS approximately 6 months after the start of the study in the study villages except the houses covered by the study interventions.

### Entomological methods.

Entomological activities included sandfly collection and sandfly density measurements at baseline (2–4 weeks) before intervention, and after interventions at 1, 3, 6, 9, and 12 months in 36 HHs in each arm. Sandfly mortality assays were performed by the WHO Cone bioassay test in 12 HHs in each arm after intervention at 1, 3, 6, 9, and 12 months.

#### Method for sandfly collection and density measurement.

Study entotechnicians collected sandflies on two consecutive nights with CDC light traps with guidance from a member of the study team. Light traps were placed in a corner of the house from 6 pm to 6 am, 2 inches away from the wall with a distance of 6 inches between the floor and the bottom of the sac. We defined sandfly density in a HH by the number of *P. argentipes* per HH for 2 nights and cluster female *P. argentipes* sandfly densities (FPAD) by the mean of the FPAD per HH in the cluster. The study entomologist performed sandfly identification following taxonomic keys by Lewis (1978, 1982)^15^^,^^16^ and Karla et al (1988).^17^

#### WHO cone bioassay test.

Study entotechnicians conducted the WHO cone bioassay test under supervision of the study entomologist using the cone method (WHOPES 2005.11)^18^ with wild caught (manually collected) sandflies at room temperature (25°–29°C) and humidity (75–85%). Study entotechnicians collected resting sandflies using manual aspirators (John W. Hock Company) in the early morning and evening. Ten to 12 female sandflies were introduced in each of the cones placed against a treated wall with IWP, IRS and untreated (control) for 30 minutes and 3 min for DWL and ITN and plain paper (control). Sandflies were then transferred from each cone to a paper cup for 24 hours of observation for calculation of mortality rates. Sandfly mortality rates were corrected using Abbot’s formula for further analysis (WHOPES 2005.11).^18^

#### Measurement of efficacy of IWP relative to DWL, ITN, and IRS.

The efficacy of IWP was determined by 1) the percent reduction of sandfly density by IWP compared with DWL, ITN, and IRS; 2) percent sandfly mortality by IWP, DWL, ITN, and IRS assessed by the WHO cone bioassay test.

### Sample size calculation.

We calculated sample size assuming 55% of sandfly density reduction compared with that by DWL, ITN, and IRS, anticipating a study power of 80% with 5% level of significance. The required number of HH was 32 per intervention. We included 36 HHs per intervention in alignment, with the sample size calculation.

### Statistical analysis.

The data management assistants entered data into Epi Info Version 3.5. Before data analysis, variables related to HHs’ socioeconomic demographics, presence of domestic animals, and protection practice from sandfly and mosquito bites were dichotomized. Household asset scores were generated by principal component analysis (PCA) using the following variables: electricity, television, khat/choki, mattress, bednet, motorcycle, bicycle, rickshaw, shallow machine, dheki, crushing mill, fishing net, store, chair/table, mobile phone, clock, almirah, researve shari, sewing machine, and fishing hatchery. Initially, we categorized PCA assets score into tertile: low, medium, and high. Then we merged medium and high into one category as “medium/high.” A data analyst checked the nature of the data by descriptive analysis, used parametric and nonparametric methods where applicable for comparing between means and performed a chi-square test to compare between proportions.

We investigated the association of the cluster FPAD with baseline characteristics of HHs in each cluster. Baseline and follow-up FPAD of the cluster were plotted to investigate the trend of the FPAD in the cluster. The FPAD of the cluster at each time point during follow-up was compared with its baseline FPAD to calculate percent reduction in FPAD in the cluster during follow-ups.

We found that baseline sandfly density of IWP cluster significantly varied from other clusters. Some of the covariates also significantly varied between IWP and other clusters. Hence, we used the difference-in-difference (DID) regression model to account the baseline differences of sandfly density as well as the variation in the covariates between IWP and other clusters during measurement of the efficacy of the IWP relative to other intervention clusters. An interaction term for the intervention arm at follow-up was included in this model to estimate the effect of the intervention. Technically the simple regression model had the following structure:

Number of female  P.argentipes=Intercept+a*Treatment+b*Time+c*Interaction+error

where treatment is one if it is the IWP and zero if it is the DWL/ITN/IRS; where time is one if follow-up and zero if baseline; and where interaction (Treatment × Time) is one if the it is the IWP group at follow-up. The following variables were controlled for in the adjusted model: occupation of HH head, HH asset, having a goat in the HH for modeling IWP against DWL; having a cattle shed in the HH, having a cow in the HH, use of mosquito coil for modeling IWP against ITN; and education of HH head, having cattle shed in the HH, and having a cow in the HH for modeling IWP against IRS.

Intervention effect was measured using DID in the average FPAD generated from the c-coefficient in the model. A negative c-coefficient means the sandfly density has decreased and hence the IWP was more effective than the comparator, and vice versa. The c-coefficient should be zero if there is no difference in the effect on sandfly density between IWP and the comparator intervention. Percentage reduction of FPAD by IWP relative to DWL, ITN, and IRS is calculated and given with 95% confidence interval (CI):

Percentage reduction of FPAD by IWP=(c−coefficient/baseline FPAD of IWP)*100

We considered an intervention effective for killing sandflies if the Abbot’s corrected sandfly mortality remained ≥ 80%. We performed all analysis by STATA version 13.0.

## RESULTS

### Association between baseline *P. argentipes* densities and HH characteristics.

The study included 217 HHs with 56, 55, 52, and 54 in the IWP, DWL, ITN, and IRS clusters, respectively, with a total population of 1,020 people. The baseline mean (95% CI) FPAD in IWP, DWL, ITN, and IRS was 1.64 (1.01–2.27), 0.56 (0.19–0.92), 0.58 (0.22–0.95), and 1.03 (0.48–1.57), respectively. The IWP FPAD differed significantly with that of DWL (*P* = 0.001) and ITN (*P* = 0.002) clusters.

The only associations between FPAD and HHs socioeconomic demographics were a negative association with a cement floor in the HH was observed in the IWP cluster, and a positive association with mud walls and regular use of bednets was observed in the IRS cluster (Table 1).

**Table 1:** Association of sociodemographic characteristics of cluster with its baseline FPAD

	IWP		*P* value	DWL		*P* value	ITN		*P* value	IRS		*P* value
	% (*n*) *N* = 36	FPAD Mean (95% CI)		% (*n*) *N* = 36	FPAD Mean (95% CI)		% (*n*) *N* = 36	FPAD Mean (95% CI)		% (n) *N* = 36	FPAD Mean (95% CI)	
Non labor HHH	69.4 (25)	1.44 (0.66, 2.22)	0.161	88.9 (32)	0.59 (0.19, 1.00)	0.687	83.3 (30)	0.67 (0.24, 1.09)	0.361	77.8 (28)	1.18 (0.50, 1.86)	0.343
Labor HHH	30.6 (11)	2.09 (1.01, 3.17)		11.1 (4)	0.25 (–0.26, 0.76)		16.7 (6)	0.17 (–0.17, 0.51)		22.2 (8)	0.50 (–0.04, 1.04)	
Literate HHH	55.6 (20)	1.65 (0.87, 2.43)	0.777	38.9 (14)	0.79 (0.04, 1.53)	0.264	50.0 (18)	0.67 (0.12, 1.21)	0.683	27.8 (10)	1.00 (–0.39, 2.39)	0.411
Illiterate HHH	44.4 (16)	1.63 (0.56, 2.69)		61.1 (22)	0.41 (0.04, 0.78)		50.0 (18)	0.50 (0.00, 1.00)		72.2 (26)	1.04 (0.48, 1.60)	
Bedrooms < 2	30.6 (11)	2.09 (1.05, 3.13)	0.141	52.8 (19)	0.58 (0.16, 1.00)	0.447	41.7 (15)	0.60 (–0.05, 1.25)	0.767	33.3 (12)	1.42 (0.23, 2.60)	0.336
Rooms ≥ 2	69.4 (25)	1.44 (0.65, 2.23)		47.2 (17)	0.53 (–0.10, 1.16)		58.3 (21)	0.57 (0.14, 1.00)		66.7 (24)	0.83 (0.26, 1.40)	
Veranda	19.4 (7)	0.71 (–0.02, 1.44)	0.127	5.6 (2)	2.50 (–2.58, 7.58)	0.308	36.1 (13)	0.23 (–0.02, 0.48)	0.311	36.1 (13)	1.08 (0.12, 2.04)	0.986
No veranda	80.6 (29)	1.86 (1.12, 2.61)		94.4 (34)	0.44 (0.17, 0.71)		63.9 (23)	0.78 (0.24, 1.32)		63.9 (23)	1.00 (0.32, 1.68)	
Cattle shed	30.6 (11)	1.64 (–0.08, 3.35)	0.215	27.8 (10)	0.80 (–0.24, 1.84)	0.879	61.1 (22)	0.64 (0.22, 1.05)	0.188	58.3 (21)	1.14 (0.26, 2.02)	0.602
No cattle shed	69.4 (25)	1.64 (1.09, 2.19)		72.2 (26)	0.46 (0.14, 0.78)		38.9 (14)	0.50 (–0.20, 1.20)		41.7 (15)	0.87 (0.39, 1.35)	
Non–mud wall	80.6 (29)	1.66 (0.91, 2.40)	0.786	97.2 (35)	0.57 (0.20, 0.95)	0.515	97.2 (35)	0.60 (0.23, 0.97)	0.515	83.3 (30)	0.60 (0.30, 0.90)	0.009
Mud wall	19.4 (7)	1.57 (0.50, 2.64)		2.8 (1)	0.00		2.8 (1)	0.00		16.7 (6)	3.17 (0.86, 5.48)	
No crack in wall	80.6 (29)	1.66 (0.91, 2.40)	0.786	97.2 (35)	0.57 (0.20, 0.95)	0.515	100.0 (36)	0.58 (0.22, 0.95)	–	97.2 (35)	0.97 (0.42, 1.52)	0.118
Crack in wall	19.4 (7)	1.57 (0.50, 2.64)		2.8 (1)	0.00		0.0 (0)			2.8 (1)	3.00	
Cement floor	16.7 (6)	0.33 (–0.34, 1.01)	0.022	2.8 (1)	0.00	0.515	2.8 (1)	0.00	0.515	16.7 (6)	0.33 (–0.09, 0.76)	0.232
Mud floor	83.3 (30)	1.90 (1.19, 2.61)		97.2 (35)	0.57 (0.20, 0.95)		97.2 (35)	0.60 (0.23, 0.97)		83.3 (30)	1.17 (0.53, 1.81)	
Dry floor	86.1 (31)	1.52 (0.80, 2.23)	0.069	94.4 (34)	0.59 (0.20, 0.97)	0.350	86.1 (31)	0.52 (0.17, 0.87)	0.536	94.4 (34)	0.97 (0.41, 1.54)	0.135
Damp floor	13.9 (5)	2.40 (1.59, 3.21)		5.6 (2)	0.00		13.9 (5)	1.00 (–0.57, 2.57)		5.6 (2)	2.00 (–0.03, 4.03)	
Low asset	27.8 (10)	2.20 (0.92, 3.48)	0.230	66.7 (24)	0.54 (0.20, 0.89)	0.342	16.7 (6)	0.67 (–0.34, 1.67)	0.855	44.4 (16)	0.56 (0.19, 0.93)	0.270
Medium/high asset	72.2 (26)	1.42 (0.70, 2.15)		33.3 (12)	0.58 (–0.30, 1.47)		83.3 (30)	0.57 (0.17, 0.96)		55.6 (20)	1.40 (0.49, 2.31)	
Cow in HH	30.6 (11)	1.91 (0.26, 3.56)	0.774	41.7 (15)	0.87 (0.13, 1.60)	0.214	58.3 (21)	0.62 (0.19, 1.05)	0.364	55.6 (20)	1.10 (0.18, 2.02)	0.352
No cow in HH	69.4 (25)	1.52 (0.94, 2.10)		58.3 (21)	0.33 (0.01, 0.66)		41.7 (15)	0.53 (–0.12, 1.19)		44.4 (16)	0.94 (0.47, 1.41)	
Goat in HH	22.2 (8)	2.75 (0.62, 4.88)	0.301	0.0 (0)		–	13.9 (5)	1.20 (–0.42, 2.82)	0.414	25.0 (9)	1.78 (–0.07, 3.62)	0.607
No goat	77.8 (28)	1.32 (0.81, 1.83)		100.0(36)	0.56 (0.19, 0.92)		86.1 (31)	0.48 (0.15, 0.82)		75.0 (27)	0.78 (0.40, 1.16)	
Chicken in HH	77.8 (28)	1.68 (0.92, 2.44)	0.952	72.2 (26)	0.65 (0.20, 1.10)	0.152	83.3 (30)	0.60 (0.20, 1.00)	0.531	86.1 (31)	1.10 (0.47, 1.72)	0.882
No chicken	22.2 (8)	1.50 (0.41, 2.59)		27.8 (10)	0.30 (–0.31, 0.91)		16.7 (6)	0.50 (–0.52, 1.52)		13.9 (5)	0.60 (0.10, 1.10)	
Duck in HH	47.2 (17)	1.82 (0.76, 2.89)	0.715	38.9 (14)	0.79 (0.01, 1.56)	0.497	41.7 (15)	0.27 (–0.04, 0.58)	0.199	25.0 (9)	1.33 (–0.18, 2.85)	0.737
No duck	52.8 (19)	1.47 (0.72, 2.22)		61.1 (22)	0.41 (0.06, 0.75)		58.3 (21)	0.81 (0.24, 1.38)		75.0 (27)	0.93 (0.38, 1.47)	
< 2 bednets	41.7 (15)	2.07 (0.89, 3.25)	0.290	52.8 (19)	0.58 (0.16, 1.00)	0.447	47.2 (17)	0.47 (0.04, 0.90)	0.785	63.9 (23)	1.00 (0.34, 1.66)	0.873
≥ 2 bednets	58.3 (21)	1.33 (0.66, 2.01)		47.2 (17)	0.53 (–0.10, 1.16)		52.8 (19)	0.68 (0.10, 1.27)		36.1 (13)	1.08 (0.09, 2.06)	
Regular bednet use	58.3 (21)	1.71 (0.93, 2.50)	0.638	83.3 (30)	0.33 (0.11, 0.56)	0.095	72.2 (26)	0.50 (0.06, 0.94)	0.171	13.9 (5)	2.40 (0.31, 4.49)	0.050
Nonregular bednet use	41.7 (15)	1.53 (0.45, 2.62)		16.7 (6)	1.67 (–0.05, 3.38)		27.8 (10)	0.80 (0.14, 1.46)		86.1 (31)	0.81 (0.30, 1.32)	
Use of mosquito coil	13.9 (5)	0.80 (–0.19, 1.79)	0.282	5.6 (2)	0.00	0.350	41.7 (15)	0.60 (–0.05, 1.25)	0.767	16.7 (6)	0.17 (–0.17, 0.51)	0.066
No mosquito coil use	86.1 (31)	1.77 (1.07, 2.48)		94.4 (34)	0.59 (0.20, 0.97)		58.3 (21)	0.57 (0.14, 1.00)		83.3 (30)	1.20 (0.57, 1.83)	

DWL = durable wall lining containing deltamethrin; FPAD = female *Phlebotomus argentipes* density; HH = household; HHH = household head; IRS = indoor residual spraying with deltamethrin; ITN = impregnation of existing bednets with KO TAB 1-2-3; IWP = insecticidal wall painting.

### FPAD and its trend during follow-up in each intervention cluster.

**Figure 2.**
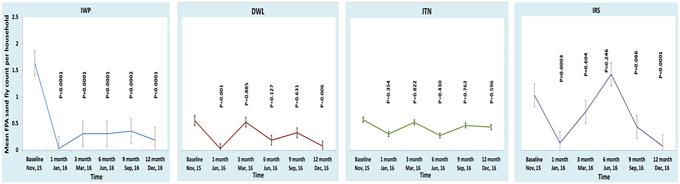
Female *Phlebotomus argentipes* sandfly density (FPAD) at baseline and during follow-up within cluster. DWL = durable wall lining containing deltamethrin; IRS = indoor residual spraying with deltamethrin; ITN = impregnation of existing bednets with KO TAB 1-2-3; IWP = insecticidal wall painting. This figure appears in color at www.ajtmh.org.

**Figure 3.**
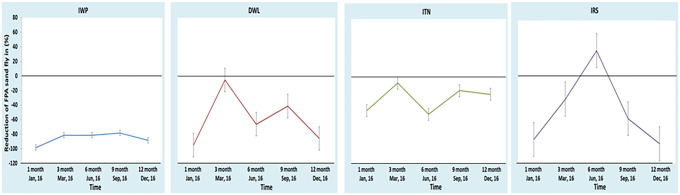
Percent reduction of female *Phlebotomus argentipes* sandfly density (FPAD) within cluster during follow-up compared with baseline FPAD of the cluster. DWL = durable wall lining containing deltamethrin; IRS = indoor residual spraying with deltamethrin; ITN = impregnation of existing bednets with KO TAB 1-2-3; IWP = insecticidal wall painting. This figure appears in color at www.ajtmh.org.

Figure 2 shows the trend of the FPAD in each intervention. The FPAD of the IWP remained significantly lower at all the time points during follow-up compared with its baseline (Figure 2). In comparison, the FPAD of the DWL and IRS clusters was significantly lower only at 1 and 12 months compared with baseline. The FPAD in the ITN cluster did not differ significantly over time compared with baseline. When considering the percentage FPAD reduction within a cluster during follow-up compared with the baseline, the only cluster in which it remained consistently lower was the IWP (Figure 3). In the other clusters, the percentage FPAD reduction was present except at month 3 in the DWL and ITN clusters and at month 6 in the IRS cluster.

### Efficacy of the IWP intervention compared with other interventions.

**Figure 4.**
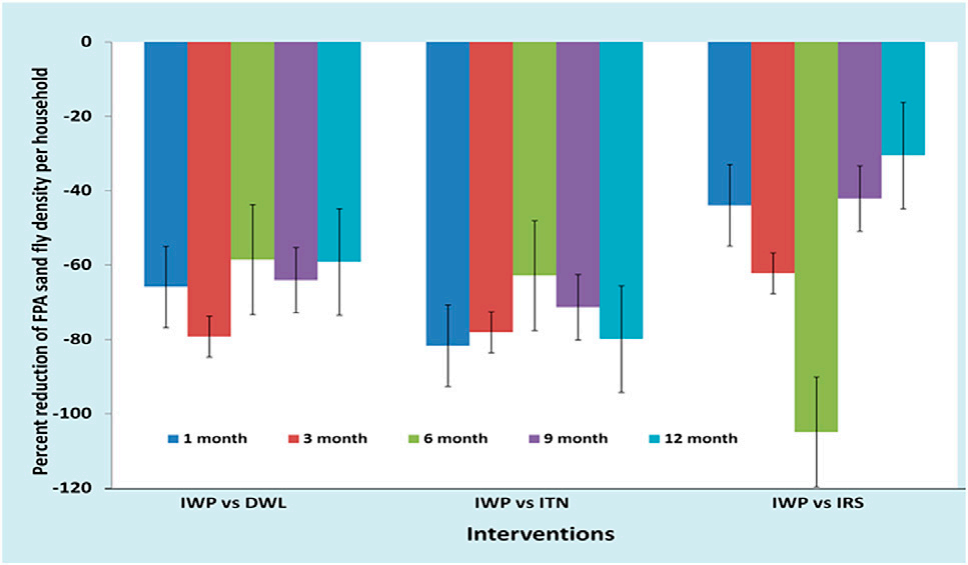
Efficacy of IWP versus other interventions for female *Phlebotomus argentipes* density (FPAD) reduction during follow-up. DWL = durable wall lining containing deltamethrin; IRS = indoor residual spraying with deltamethrin; ITN = impregnation of existing bednets with KO TAB 1-2-3; IWP = insecticidal wall painting.

The IWP had a statistically significant higher baseline FPAD compared with the DWL and ITN. The IWP was also significantly different from other clusters regarding HH head occupation, ownership of cattle shed, HH asset score, ownership of domestic animals and bednets, and use of mosquito coil, for example (Table 2). We therefore selected the difference-in-difference (DID) regression model to measure the efficacy of the IWP intervention relative to other interventions to adjust the effect of the significant covariates. Using DID, we found that IWP had better efficacy for FPAD reduction throughout the follow-up period relative to DWL, ITN, and IRS (Figure 4). The efficacy of IWP FPAD reduction varied from –59% to –91% against DWL, –62% to –82% against ITN, and –30% to –117% against IRS across the different follow-up times (Table 3).

**Table 2:** Comparison of sociodemographic characteristics between IWP and other clusters

	IWP	DWL	ITN	IRS	*P* value	*P* value	*P* value	
Variables	% (*n*), *N* = 36	% (*n*), *N* = 36	% (*n*), *N* = 36	% (*n*), *N* = 36	IWP vs. DWL	IWP vs. ITN	IWP vs. IRS	
Labor HHH	30.56 (11)	11.11 (4)	16.67 (6)	22.2 (8)	0.042	0.165	0.422
Illiterate HHH	44.44 (16)	61.11 (22)	50.00 (18)	72.2 (26)	0.157	0.637	0.017
< 2 bedrooms	30.6 (11)	52.8 (19)	41.7 (15)	33.3 (12)	0.056	0.326	0.800
Veranda in HH	19.44 (7)	5.56 (2)	36.11 (13)	36.1 (13)	0.151	0.114	0.114
Cattle shed in HH	30.56 (11)	27.78 (10)	61.11 (22)	58.3 (21)	0.795	0.009	0.018
Low asset	27.8 (10)	66.7 (24)	16.7 (6)	44.4 (16)	0.001	0.257	0.141
Cow in the HH	30.56 (11)	41.67 (15)	58.33 (21)	55.6 (20)	0.326	0.018	0.032
Goat in the HH	22.22 (8)	0.0 (0)	13.89 (5)	25.0 (9)	0.005	0.358	0.781
Chicken in the HH	77.78 (28)	72.22 (26)	83.33 (30)	86.1 (31)	0.586	0.551	0.358
Duck in the HH	47.22 (17)	38.89 (14)	41.67 (15)	25.0 (9)	0.475	0.635	0.050
< 2 bednets	41.7 (15)	52.8 (19)	47.2 (17)	63.9 (23)	0.345	0.635	0.059
Use of mosquito coil	13.89 (5)	5.56 (2)	41.67 (15)	16.7 (6)	0.429	0.009	0.743
Baseline FPAD mean (95% CI)	1.64 (1.01, 2.27)	0.56 (0.19, 0.92)	0.58 (0.22, 0.95)	1.03 (0.48, 1.57)	0.001	0.002	0.076

CI = confidence interval; DWL = durable wall lining containing deltamethrin; FPAD = female *Phlebotomus argentipes* density; HH = household; HHH = household head; IRS = indoor residual spraying with deltamethrin; ITN = impregnation of existing bednets with KO TAB 1-2-3; IWP = insecticidal wall painting.

**Table 3:** Female *Phlemotomus argentipes* sandfly per household and their comparison between IWP versus other arms at baseline and follow-up

					Percentage reduction of sandfly densities by IWP relative to DWL, ITN, and IRS						
Time	Female *P. argentipes* sandfly per household Mean (95% CI)				IWP vs. DWL			IWP vs. ITN		IWP vs. IRS	
	IWP (*N* = 36)	DWL (*N* = 36)	ITN (*N* = 36)	IRS (*N* = 36)	Unadjusted	Adjusted*	Unadjusted		Adjusted^†^	Unadjusted	Adjusted^‡^
Baseline	1.64 (1.01, 2.27)	0.56 (0.19, 0.92)	0.58 (0.22, 0.95)	1.03(0.48, 1.57)	–	–	–		–	–	–
1-month follow-up	0.03 (–0.03, 0.08)	0.03 (–0.03, 0.08)	0.31 (0.09, 0.52)	0.14 (–0.01, 0.28)	−65.85 (–109.76, −22.56)	−65.85 (–109.15, −22.56)	−81.10 (–126.83, −35.98)		−81.10 (–126.83, −35.37)	−43.90 (–94.51, 6.10)	−43.90 (–95.12, 6.71)
3-month follow-up	0.31 (0.11, 0.50)	0.53 (0.21, 0.85)	0.53 (0.19, 0.87)	0.72 (0.39, 1.05)	−79.88 (–128.05, −31.10)	−79.88 (–127.44, −31.71)	−78.05 (–126.83, −28.66)		−78.05 (–126.83, −28.66)	−62.80 (–117.07, −7.93)	−62.80 (–117.68, −7.32)
6-month follow-up	0.31 (0.13, 0.48)	0.19 (0.04, 0.35)	0.28 (0.10, 0.45)	1.42 (0.79, 2.05)	−59.15 (–104.88, −13.41)	−59.15 (–104.88, −14.02)	−62.80 (–108.54, −17.07)		−62.80 (–109.15, −16.46)	−104.88 (–167.68, −42.07)	−104.88 (–168.29, −42.07)
9-month follow-up	0.36 (0.12, 0.61)	0.33 (0.14, 0.53)	0.47 (0.18, 0.77)	0.44 (0.17, 0.72)	−64.63 (–111.59, −17.07)	−64.63 (–111.59, −17.07)	−71.34 (–120.12, −21.95)		−71.34 (–120.73, −21.34)	−42.07 (–96.34, 11.59)	−42.07 (–96.95, 12.20)
12-month follow-up	0.19 (0.06, 0.33)	0.08 (–0.04, 0.21)	0.44 (0.15, 0.74)	0.08 (–0.01, 0.18)	−59.15 (–104.27, −14.63)	−59.15 (–103.66, −15.24)	−79.88 (–126.83, −32.32)		−79.88 (–127.44, −31.71)	−30.49 (–81.10, 20.12)	−30.49 (–81.71, 20.73)

CI = confidence interval; DWL = durable wall lining containing deltamethrin; IRS = indoor residual spraying with deltamethrin; ITN = impregnation of existing bednets with KO TAB 1-2-3; IWP = insecticidal wall painting.

* Adjusted by the covariates occupation of household head, household assets, and having goat in the household.

^†^ Adjusted by the covariates having cattle shed in the household, having cow in the household, and use of mosquito coil.

^‡^ Adjusted by the covariates education of household head, having cattle shed in the household, and having cow in the household.

### Abbot’s corrected sandfly mortality by IWP, DWL, ITN, and IRS.

**Figure 5.**
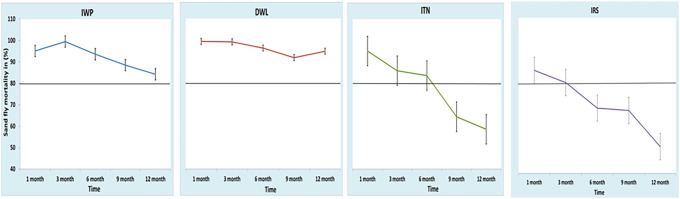
Abbot-corrected *Phlebotomus argentipes* sandfly mortality by interventions and follow-up. DWL = durable wall lining containing deltamethrin; IRS = indoor residual spraying with deltamethrin; ITN = impregnation of existing bednets with KO TAB 1-2-3; IWP = insecticidal wall painting. This figure appears in color at www.ajtmh.org.

Sandfly mortality by IWP was comparable to that by DWL, ranging from 84% to 95% during follow-up and was higher than that by ITN and IRS (Table 4). Using a cutoff for efficacy for sandfly mortality of ≥ 80%, IWP and DWL were consistently effective throughout the 12-month follow-up, whereas ITN and IRS were effective for 6 and 3 months, respectively (Figure 5).

**Table 4:** Abbot-corrected *Phlemotomus argentipes* sandfly mortality by interventions and follow-up

	Average corrected *P. argentipes* sandfly mortality (95% CI)			
Time	IWP	DWL	ITN	IRS
1-month follow-up	95.12% (91.54–98.70%)	99.31% (97.78–100.83%)	95.08% (93.41–96.76%)	86.36% (82.45–90.26%)
3-month follow-up	99.50% (98.74–100.25%)	99.11% (97.78–100.44%)	85.90% (82.36–89.43%)	80.87% (77.35–84.38%)
6-month follow-up	93.60% (90.25–96.94%)	96.22% (93.84–98.61%)	83.64% (80.26–87.01%)	69.36% (64.0–74.71%)
9-month follow-up	88.52% (84.09–92.94%)	91.77% (89.46–94.07%)	64.43% (59.80–69.06%)	68.33% (64.79–71.86%)
12-month follow-up	84.24% (80.86–87.62%)	94.75% (91.79–97.72%)	58.57% (52.31–64.83%)	52.05% (46.21–57.89%)

DWL = durable wall lining containing deltamethrin; IRS = indoor residual spraying with deltamethrin; ITN = impregnation of existing bednets with KO TAB 1-2-3; IWP = insecticidal wall painting.

### Safety and acceptability.

All four interventions IWP, DWL, ITN, and IRS were highly safe, with no observed serious adverse events or adverse reactions. Twelve percent of HH heads in the ITN cluster perceived a transient unpleasant smell. Ninety-four percent, 98%, 98%, and 54% of HHs in the IWP, DWL, ITN, and IRS groups, respectively, liked the intervention. Direct observation over 12 months found that IWP and DWL were physically intact in 98% and 100% of HHs, respectively. The occupants of 90%, 80%, 54%, and 29% of the HHs that received IWP, DWL, ITN, and IRS, respectively, perceived a decrease in mosquito and sandfly presence in the house.

## DISCUSSION

The major findings of the study are that all vector control interventions including ITN, DWL (1-meter high), IWP, and IRS are effective for sandfly control; however, the strength and duration of efficacy differed considerably, and the IWP had superior performance compared with ITN, DWL, and IRS interventions.

The conventional method for sandfly control is IRS, which has been used by the NKEP in Bangladesh since 2012. Its efficacy has been demonstrated by many previous studies.^6^^,^^7^ This study confirmed that IRS is still effective in Bangladesh, but its duration of efficacy is short (approximately 4.5 months); thereafter, the sandfly density increases again. The decline after the peak observed in this study perhaps was due to seasonality (November–February are typically months in which sandfly densities decline). IRS may not be sustainable in the long run due to the cost and complexity and short-term efficacy requiring regular treatments, compounded with a decreased acceptability by communities, as shown in this and previous studies.^19^^,^^20^ These drawbacks of IRS urge the identification of cost-effective alternatives with longer duration of efficacy that can be taken up by the community. IWP, DWL, and ITN could be such potential methods for sandfly control.

Studies in the past on the Indian subcontinent including Bangladesh demonstrated the high efficacy of DWL (for both full- and reduced-surface coverage) for controlling sandflies.^11^^,^^21^ In this study, we provide further evidence of the efficacy of reduced-surface coverage DWL because the vector density remained low for 12 months, and the sandfly killing capacity was marginally higher than that of IWP. This is because DWL has the highest concentration of insecticides among all existing vector control tools. We therefore consider DWL an attractive vector control tool if a national program and/or community can afford it or if production can be locally owned. Involvement of local microcredit systems for financial support for the community may help make DWL affordable.

In a previous study, we found that ITN was effective for controlling sandflies for up to18 months,^8^ whereas in this study, it was only 6 months. This discrepancy can be explained by the different study designs. In the previous study, mass bednet impregnation was performed in which hundreds of HHs and thousands of existing bednets were impregnated, resulting in a large spatial effect; this did not happen in our study, where only approximately 50 HHs were treated in a village. In India and Nepal, the reduction in vector densities did not translate into a reduction of VL cases, whereas in Bangladesh, mass bednet impregnation programs reduced both vector densities and VL cases.^8^^–^^10^ Therefore, bednet impregnations with slow-release insecticide tablets are other options for sandfly control in Bangladesh.

IWP containing three insecticides is a new tool for vector control and was the intervention of interest in this study. Although it was developed and had been used for pest control, studies in Africa have demonstrated its usefulness for controlling *Anopheles*, the malaria vector, a finding that encouraged us to investigate its efficacy on sandflies.^12^^,^^13^ Moreover, a recent study from Nepal found IWP effective for sandfly control.^14^ In this study, we compared its efficacy against DWL, ITN, and IRS and found IWP to be the most powerful tool for sandfly density control because it maintained a low sandfly density for a longer period and has comparable sandfly killing effects to DWL. The IWP cluster differed from other clusters regarding some covariates (e.g., having a cattle shed in the HH, animals such as a cow or goat in the HH, use of bednets, use of mosquito coil). We minimized their potential impact on sandfly density using the DID regression model during comparison of the relative efficacy of IWP against the other interventions. It will be interesting to have longer term results to determine how the insecticide capacity of the tools evolve with time, and hence how often the intervention must be renewed.

The safety of IWP, DWL, ITN, and IRS were excellent because no serious adverse events or adverse reactions were reported, and no inconveniences were reported except transient unpleasant smell in 12% of the HHs where ITN were deployed. IRS acceptability, however, is a concern, as it decreased to 54%. This underlines the need of sandfly control tool(s) that can be acceptable and taken up by the community people by themselves. Potential options are IWP, ITN, and DWL. Currently, manufacturers have stopped production of the KO TAB 1-2-3 and DWL, and therefore IWP remains the only available option at present.

The main limitation of the study is that it was not possible to investigate the impact of the interventions on VL cases because the current disease burden in Bangladesh is fortunately low. We also could not measure the concentration of the insecticides and their decay over time in ITN, DWL, and IWP because we did not have the appropriate technology. We also could not include an untreated control cluster because the control program rolled out the routine IRS cycle during follow-up phase of this study. Theoretically, this could have cointervention effects. However, the differences found in the effects of the different interventions on FPAD and its varying trends in different clusters over time indicate that the potential spatial effect, cointervention effect, and contamination of the routine IRS did not compromise our study results and conclusions.

We now have evidence for a range of effective sandfly control tools. On the basis of our study, we consider IWP and DWL superior to IRS in terms of strength and duration of efficacy and long-term sustainability for sandfly control. Of the two, IWP is available and can be taken up by communities. A study is underway to investigate the efficacy of IWP for sandfly control deployed by the community under supervision of public health workers.

The current price of IWP (30 USD per HH) and DWL (50 USD per HH) for full coverage is comparatively high for communities and national programs. The partial coverage with DWL makes its cost closer to that of IWP. The IWP and DWL interventions may offer options for local entrepreneurship for producing and applying the materials. Transfer of production to local manufacturers that can meet quality standards will reduce their price. This process has already started in Bangladesh where the IWP is now produced locally under supervision of the original manufacturer. Future studies are needed with locally produced IWP for investigation of its cost-effectiveness for sandfly and other vectors control. It would also be useful to have data in the longer term to show how the efficacy for sandfly control evolves over time beyond 12 months. For this reason, we have an ongoing study for long-term sustainability.

In summary, IWP is a new effective tool for VL vector control. On the basis of our study evidence and the current sandfly control method by the NKEP (IRS in 50–60 HHs around a VL index case), the NKEP may consider IWP for sandfly control for subsequent phases of the program where a longer duration of efficacy is required.

## References

[1] Bern C Chowdhury R, 2006. The epidemiology of visceral leishmaniasis in Bangladesh: prospects for improved control. Indian J Med Res 123: 275–288. 16778310

[2] Singh OP Hasker E Boelaert M Sundar S, 2016. Elimination of visceral leishmaniasis on the Indian sub-continent. Lancet Infect Dis 16: e304–e309. 27692643 10.1016/S1473-3099(16)30140-2 PMC5177523

[3] Rijal S Sundar S Mondal D Das P Alvar J Boelaert M, 2019. Eliminating visceral leishmaniasis in South Asia: the road ahead. BMJ 364: k5224. 30670453 10.1136/bmj.k5224 PMC6340338

[4] WHO , Regional Technical Advisory Group on Kala-azar Elimination, 2005. *Report of the First Meeting, Manesar, Haryana, 20–23 December 2004.* New Delhi, India: Regional Office for South-East Asia.

[5] World Health Organization , Regional Office for South-East Asia, 2020. *Report of Meeting of the Regional Technical Advisory Group (RTAG) on Visceral Leishmaniasis and the National Visceral Leishmaniasis Programme Managers of Endemic Member States.* WHO Regional Office for South-East Asia. License: CC BY-NC-SA 3.0 IGO. Available at: https://apps.who.int/iris/handle/10665/340612.

[6] Joshi AB , 2009. Chemical and environmental vector control as a contribution to the elimination of visceral leishmaniasis on the Indian subcontinent: cluster randomized controlled trials in Bangladesh, India and Nepal. BMC Med 7: 54. 19804620 10.1186/1741-7015-7-54 PMC2763005

[7] Chowdhury R , 2017. Control of *Phlebotomus argentipes* (Diptera: Psychodidae) sandfly in Bangladesh: a cluster randomized controlled trial. PLoS Negl Trop Dis 11: e0005890. 28873425 10.1371/journal.pntd.0005890 PMC5600390

[8] Mondal D Huda MM Karmoker MK Ghosh D Matlashewski G Nabi SG Kroeger A, 2013. Reducing visceral leishmaniasis by insecticide impregnation of bed-nets, Bangladesh. Emerg Infect Dis 19: 1131–1134. 23764246 10.3201/eid1907.120932 PMC3713966

[9] Chowdhury R , 2019. Effect of insecticide-treated bednets on visceral leishmaniasis incidence in Bangladesh. A retrospective cohort analysis. PLoS Negl Trop Dis 13: e0007724. 31525195 10.1371/journal.pntd.0007724 PMC6762203

[10] Picado A , 2010. Longlasting insecticidal nets for prevention of *Leishmania donovani* infection in India and Nepal: paired cluster randomised trial. BMJ 341: c6760. 21190965 10.1136/bmj.c6760 PMC3011370

[11] Mondal D , 2016. Efficacy, safety and cost of insecticide treated wall lining, insecticide treated bednets and indoor wall wash with lime for visceral leishmaniasis vector control in the Indian subcontinent: a multi-country cluster randomized controlled trial. PLoS Negl Trop Dis 10: e0004932. 27533097 10.1371/journal.pntd.0004932 PMC4988640

[12] Mosqueira B , 2015. Pilot study on the combination of an organophosphate-based insecticide paint and pyrethroid-treated long lasting nets against pyrethroid resistant malaria vectors in Burkina Faso. Acta Trop 148:162–169. 25959771 10.1016/j.actatropica.2015.04.010

[13] Mosqueira B Chabi J Chandre F Akogbeto M Hougard JM Carnevale P Mas-Coma S, 2010. Efficacy of an insecticide paint against malaria vectors and nuisance in West Africa—part 2: field evaluation. Malar J 9: 341. 21108820 10.1186/1475-2875-9-341 PMC3004939

[14] Banjara MR Das ML Gurung CK Singh VK Joshi AB Matlashewski G Kroeger A Olliaro P, 2019. Integrating case detection of visceral leishmaniasis and other febrile illness with vector control in the post-elimination phase in Nepal. Am J Trop Med Hyg 100: 108–114. 30426921 10.4269/ajtmh.18-0307 PMC6335889

[15] Lewis DJ, 1978. The phlebotomine sandflies (Diptera: Psychodidae) of the Oriental region. Bulletin of the British Museum (Natural History), B. Entomology. 37: 217–343.

[16] Lewis DJ, 1982. A taxonomic review of the genus *Phlebotomus* (Diptera: Psychodidae). Bull Br Mus 45: 121–209.

[17] Kalra NL Bang YH, 1988. *Manual on Entomology in Visceral Leishmaniasis (SAE/VBC/35).* New Delhi, India: World Health Organization Regional Office for South East Asia, 88.

[18] World Health Organization, 2005 *Guidelines for Laboratory and Field Testing of Long-lasting Insecticidal Mosquito Nets.* Document No. WHO/CDS/WHOPES/GCDPP/2005.11.

[19] Kumar V Kesari S Dinesh DS Tiwari AK Kumar AJ Kumar R Singh VP Das P, 2009. A report on the indoor residual spraying (IRS) in the control of *Phlebotomus argentipes*, the vector of visceral leishmaniasis in Bihar (India): an initiative towards total elimination targeting 2015 (Series-1). J Vector Borne Dis 46: 225–229. 19724087

[20] Chowdhury R , 2018. Indoor residual spraying for kala-azar vector control in Bangladesh: a continuing challenge. PLoS Negl Trop Dis 12: e0006846. 30273402 10.1371/journal.pntd.0006846 PMC6181438

[21] Huda MM , 2016. Entomological efficacy of durable wall lining with reduced wall surface coverage for strengthening visceral leishmaniasis vector control in Bangladesh, India and Nepal. *BMC* Infect Dis 16: 539. 10.1186/s12879-016-1881-8 PMC5052807 27716091

